# The complete mitochondrial genome sequence and gene organization of *Lepidotrigla Kanagashira* (Scorpaeniformes, Triglidae) with phylogenetic consideration

**DOI:** 10.1080/23802359.2019.1698326

**Published:** 2019-12-13

**Authors:** Wei Wang, Ming Zhao, Linzi Zhang, Chunyan Ma, Wei Chen, Zhiqiang Liu, Fengying Zhang, Lingbo Ma

**Affiliations:** a Key Laboratory of Oceanic and Polar Fisheries, Ministry of Agriculture and Rural Affairs, East China Sea Fisheries Research Institute, Chinese Academy of Fishery Sciences, Shanghai, China;; b College of Fisheries and Life Sciences, Shanghai Ocean University, Shanghai, China

**Keywords:** *Lepidotrigla kanagashira*, mitochondrial genome, genome structure, phylogenetic tree, evolutionary relationships

## Abstract

The complete mitochondrial genome DNA sequence of *Lepidotrigla kanagashira* was 16,504 bp in length. It consists of 13 protein-coding genes, two ribosomal RNA genes, 22 transfer RNA genes and one control region. Among 22 tRNA genes, 8 tRNAs were encoded on the L-strand. The overall base composition of the genome is 26.65% for A, 25.42% for T, 30.89% for C and17.04% for G. The phylogenetic tree suggested that *L. kanagashira* was genetically closest to *L. microptera* and *Chelidonichthys kumu* among 13 related species. This study could provide some valuable information for further studies on *L. kanagashira.*

*Lepidotrigla kanagashira*, a kind of benthic and carnivorous fish, is a member of Triglidae, broadly distributes in Western Pacific: Tosa Bay, Japan and the South China Sea (Masuda et al 1984; Musick 1999; Froese and Pauly 2012). Although mitochondrial DNA plays a significant part on the studies of its population genetics, phylogenetics and evolution (Avise et al. 1984; Zhong et al. 2013; Xia et al. 2015). So far, there was no introduction about the complete mitochondrial genome of *L. kanagashira*.

The specimen of *L. kanagashira* whose Specimen Accession number is Esfri-F0094-1-SE was collected from Xiamen, Fujian Province of China in 2018. It was stored in freezing herbarium of East China Sea Fisheries Research Institute, Chinese Academy of Fishery Science. Genomic DNA was extracted from muscle tissue using Animal Genomic DNA Extraction Kit (TIANGEN, Beijing, China) according to the manufacturer’s recommended protocol. In present study, the full length of complete mitochondrial DNA of *L. kanagashira* has been sequenced by the Roche 454 Genome Sequencer FLX System (16,504 bp, GenBank accession number. MK784116). The base composition of its mitogenome is 26.65% for A, 25.42% for T, 30.89% for C and17.04% for G. The overall A + T content of the mitochondrial genome is 52.07%. The complete mitogenomic sequence obtained includes 13 protein-coding genes, two ribosomal RNA genes, 22 transfer RNA genes and one control region. 28 of these 37 genes were encoded on the heavy strand, and nine were encoded on the light strand just as in other teleosts (Song et al. 2016). The overall length of protein coding genes is 11,428 bp. Two kinds of start codons (ATG and GTG) and four types of stop codons (TAA, TAG, TA, T) were identified in 13 protein-coding genes. The length of control region (D-loop) which has a higher A + T content (59.02%) is 837 bp, and its overall nucleotide composition is 29.87% for A, 22.82% for C, 18.16% for G and 29.15% for T.

To evaluate its phylogeny and evolution, the phylogenetic tree was constructed with significant bootstrap supports based on the Neighbour-joining method using MEGA 5.1 (Kumar et al. 2008) (Figure 1). *Lateolabrax japonicus* and *Clupea harengus* were used as an out-group. The NJ tree showed that *L. kanagashira* represented the closest relationship with *Lepidotrigla microptera*, and clustered as a group, then formed a big branch together with *Chelidonichthys kumu* and *Satyrichthys amiscus*. This study will be important to the genetic conservation and the phylogenetic classification of *L. kanagashira*.

**Figure 1.**
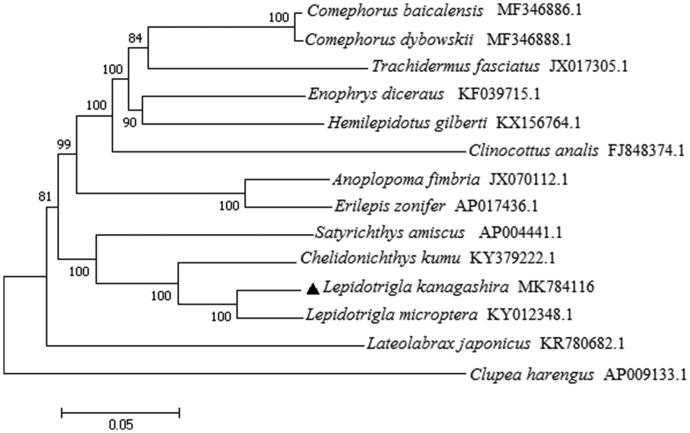
Phylogenetic position of *Lepidotrigla kanagashira* within Scorpaeniformes based on 13 protein-coding genes using neighbour-joining method. *L. kanagashira* was highlighted with a black triangle.
